# Role of Gut Microbiota in Cannabinoid-Mediated Suppression of Inflammation

**DOI:** 10.3389/adar.2022.10550

**Published:** 2022-07-14

**Authors:** Kontham Kulangara Varsha, Mitzi Nagarkatti, Prakash Nagarkatti

**Affiliations:** Department of Pathology, Microbiology and Immunology, School of Medicine, University of South Carolina, Columbia, SC, United States

**Keywords:** inflammation, cannabinoids, microbiota, drug abuse, endocannabinoids

## Abstract

Cannabinoids and the endocannabinoid system have been well established to play a crucial role in the regulation of the immune response. Also, emerging data from numerous investigations unravel the imperative role of gut microbiota and their metabolites in the maintenance of immune homeostasis and gut barrier integrity. In this review, we concisely report the immunosuppressive mechanisms triggered by cannabinoids, and how they are closely associated with the alterations in the gut microbiome and metabolome following exposure to endogenous or exogenous cannabinoids. We discuss how cannabinoid-mediated induction of microbial secondary bile acids, short chain fatty acids, and indole metabolites, produced in the gut, can suppress inflammation even in distal organs. While clearly, more clinical studies are necessary to establish the cross talk between exo- or endocannabinoid system with the gut microbiome and the immune system, the current evidence opens a new avenue of cannabinoid-gut-microbiota-based therapeutics to regulate immunological disorders.

## Introduction

*Cannabis sativa*, otherwise known as marijuana, has a rich history of being used for medical and recreational purposes. The complex biochemical metabolism of cannabis leads to the production of over 550 chemical constituents of which over 100 are identified as phytocannabinoids (1). The chemical structure of the non-psychoactive cannabinoid compound, cannabidiol (CBD) was first deduced in 1963 followed by the identification of the psychoactive cannabinoid, δ9-tertrahydrocannabinol (THC), in 1964 (2). Cannabinoids accomplish their physiological and behavioral consequences *via* their binding to the cannabinoid G-protein-coupled receptors (GPCRs), CB1 and CB2 (CBRs). The existence of these receptors and their endogenous ligands, named endocannabinoids (eCBs), in the human system was discovered in the 1990s thus revealing the functions of eCB system in neuronal and immunomodulatory functions. eCBs such as anandamide (AEA) and 2-arachydonoylglycerol (2-AG), which are native lipid-based retrograde neurotransmitters, as well as exogenous cannabinoids such as THC, act as strong agonists of CBRs. CBD, unlike THC, is not psychoactive and considered to be a negative allosteric modulator of the CB1 receptors (3). Fatty acid amide hydrolase (FAAH) and monoacylglycerol acid lipase that break down AEA and 2–AG, respectively, are the two other important participants of the eCB signaling system (4-6). The CB1 and CB2 receptors are expressed primarily in the brain and immune cells respectively and mediate nearly all the effects of both endogenous and exogenous cannabinoids (6, 7).

The downstream signaling initiated by CBRs involves the inactivation of protein kinase A following inhibition of adenyl cyclase activity and a decrease in cyclic adenosine monophosphate (cAMP) levels. In addition, CBRs trigger an array of various mechanisms in the wake of activation. This includes multiple effector protein kinase signaling cascades related to cell proliferation and survival such as phosphoinositide-3-kinase–protein kinase B/Akt (PI3K-PKB/AKT), p38 mitogen-activated protein (p38 MAP) kinases, extracellular signal-regulated kinase (ERK) as well as focal adhesion kinase (FAK) (8). Coupling to ion channels, phospholipase-cb activation, and ceramide biosynthesis are certain other pathways activated by CBRs (7–11). It is demonstrated that eCBs can also bind to non-CBRs, of which the most investigated are transient receptor potential vanilloid 1 (TRPV1) channel along with peroxisome proliferator-activated receptor and orphan GPCRs (9, 12). Most of these pathways mentioned above are entwined with maintenance of immune homeostasis. For example, certain subsets of effector CD4^+^ T cells depend on the PI3K signaling activation for their differentiation and steering of effector functions. Additionally, PI3K and p38MAPK signaling are involved in the production of inflammatory cytokines (13, 14), ERK signaling plays a role in resistance to immunomodulatory drugs (15), and nuclear translocation of FAK is important to regulate inflammatory gene expression of chemokines and cytokines (16). Such studies demonstrate that the downstream signaling pathways initiated by CBRs can lead to immunomodulation.

It is well documented that the gut microbiome plays a crucial role in host metabolism as well as the balance between pro- and anti-inflammatory responses, thereby controlling disease pathogenesis (17). Short-chain fatty acids (SCFAs), lipopolysaccharides (LPS), and other biologically active metabolites generated by various microbial species contribute toward immune regulation, activation or suppression (18). Thus, dietary and medical interventions that manipulate the composition of the microbiome have been shown to cause pro- or anti-inflammatory milieu (19). There is emerging evidence that the gut microbiome and eCB system communicate via signaling pathways involved in nutrient processing and energy metabolism (17). In this mini-review, we briefly recite an account of the effects and mechanisms of endogenous and exogenous cannabinoids on immunosuppression via microbiome-mediated activities.

## Mechanisms and Nature of Immunomodulation Caused by Cannabinoids

Cannabinoids are well established as anti-inflammatory agents with a significant and wide range of immunosuppressive properties that have been meticulously reviewed before (20–25). CBD, the nonpsychotic cannabinoid, was shown to induce myeloid-derived suppressor cells (MDSCs) which suppressed T cell proliferation *in vitro* and *in vivo* (26). MDSCs mostly express CD11b and Gr-1 and represent a heterogeneous population of immature myeloid cells which produce arginase 1 and inducible nitric oxide synthase that enables them to suppress T cell proliferation (27). Cannabinoid-induced MDSCs upon adoptive transfer were shown to attenuate LPS-induced acute inflammation *in vivo* (26, 28). The psychoactive THC was also shown to induce MDSCs independent of TLR4. THC mobilized MDSCs from bone marrow and caused their expansion in the periphery (29). In addition to the generation of MDSCs, cannabinoids have also been shown to induce regulatory T cells (Tregs) (30–33). Such T cells express FoxP3, a transcription factor that plays a critical role in their differentiation and functions, and secrete immunosuppressive cytokines such as interleukin (IL)-10 and transforming growth factor β (TGF-β) (34). Additionally, cannabinoids can also induce apoptosis of immune cells such as T and B lymphocytes, macrophages, and dendritic cells (DCs) leading to immunosuppression (35). THC triggered DC apoptosis via reduction of mitochondrial membrane permeability, cleavage of Bid, activation of caspase cascade, and release of cytochrome-c (36). THC treatment caused phosphorylation of IkappaB-alpha and augmented apoptotic gene transcription regulated by NF-kappaB (35–38). Moreover, agonists of CBRs can disrupt the balance of pro and anti-inflammatory cytokines. THC exposure restrained the production of IL2, IL-12, and interferon-gamma (IFN-γ), and altered the equilibrium of T helper 1 (Th1)/T helper 2 (Th2) cytokines in a CB2R dependent manner (39, 40). Also, THC and AEA were shown to suppress inflammatory Th1 and Th17 response during delayed-type hypersensitivity response (41, 42).

Epigenetic modulations are additional mechanisms of immunosuppression triggered by cannabinoids (43). The eCB system undergoes epigenetic modifications and such variations are observed in pathological disorders such as Diabetes, Parkinson’s, Alzheimer’s, and colorectal cancer. The main targets of these modifications are the genes CNR1 and CNR2 that encode for CB1R and CB2R along with FAAH (44–46). Recent investigations provide insights into cannabinoid-mediated epigenome modifications and their impact on the suppression of the immune system. THC treatment increased methylation of the promoter region of DNA methyl transferases, DNMT3a and DNMT3b in MDSCs leading to subsequent reduction in DNMT3a and DNMT3b expressions in C57BL/6 mice. Moreover, a decrease in the methylation of Arg1 and STAT3 promoter regions was observed that led to over-expression of Arg1 and STAT3 (47). THC was shown to activate or suppress the expression of genes via histone modifications. THC treatment led to histone modifications that led to increases in Th2 cytokine genes while suppressing Th1 cytokine genes, thereby switching the immune response from Th1 to Th2 (48).

Up-regulation or down-regulation of microRNAs (miRNAs) are another major route of epigenetic alterations prompted by cannabinoids. Treatment of C57BL/6 mice with THC elevated miR-21 while lowered miR-29b expression that was associated with a corresponding increase in SMAD7 and decrease in IFN-γ expressions. This in turn inhibited Th1/Th17 activation in delayed-type hypersensitivity reaction (47). The eCB, AEA mitigated Staphylococcal enterotoxin B (SEB)-induced acute respiratory distress syndrome (ARDS) in mice via down-regulation of miRNA-23a-3p, which up-regulated arginase and TGF-β2, and miRNA-34a-5p that prompted FoxP3 induction. A reduction in pro-inflammatory cytokines such as IL-2, TNF-α, and IFN-γ, while an increase in MDSCs was detected following AEA treatment (49). THC administration into SEB-injected C3H/HeJ mice was reported to down-regulate miR-17/92 and miR-374b/421 clusters while up-regulating miR146a leading to the release of PTEN thus acting as an AKT inhibitor leading to a reduction in IFN-γ production (31). Another study reported that THC altered expressions of the members of miR-17-92 cluster, particularly miR-18a that directed the release of PTEN (50). A detailed description of cannabinoid-mediated epigenetic modulations pertaining to immune suppression has been recently published (43). It is interesting to note that the intestinal microbiota and their metabolites have been shown to regulate several epigenetic pathways (51). This raises the question of whether the cannabinoid-mediated changes in the epigenetic pathways are linked to the gut microbiota.

## Gut Microbiota, eCB System, and Gut-Brain Axis

The diverse intestinal microbial population found in the gut shares a mutual symbiotic relationship with the host. The microbiota benefits the host by modulation of gut motility, intestinal barrier function, and nutrient absorption. Moreover, gut microbiota plays a major role in host metabolism and is associated with regulation of the inflammatory status of the host not only in the gut but also in other organs such as the brain (52, 53). Thus, alterations in the microbiota, called dysbiosis, caused by nutrition, stress, environmental factors, and drugs, can have either beneficial or deleterious effects on the inflammatory status of the host. Gut-brain axis, which is the bidirectional crosstalk between the central and enteric nervous systems is influenced by gut microbiota via neural, endocrine, and immune networks (54). Emerging data establishes the influence of gut microbiota in anxiety and depression-like behavior (55). Clinical studies denote the abundance of pro-inflammatory and reduced SCFA producing bacterial species in these disease conditions, and this pathophysiology relates to the transmission of peripheral inflammation to the brain (56). There are recent reviews which have discussed the effects of cannabinoids, including CBD and alcohol in the microbiota-gut-brain axis (57) and therefore not further discussed this topic in this review.

It has been widely recognized that eCB is dynamically involved in the regulation of glucose and energy metabolism. It is also important to note that the immunomodulatory effects of eCBs are not always mediated via CBRs. Metabolism of 2-AG and AEA generates lipid components and hence acts as a source of arachidonic acid in the biosynthesis of additional pro-inflammatory lipids (58). Advanced research in the field of eCB system and immune modulation indicates the contribution of bio lipid members of eCB system in the onset or progression of various diseases such as obesity, diabetes, inflammatory bowel disease (IBD), and multiple sclerosis (MS) which are also reported to be augmented by alterations in the microbiota (59). Elevated eCBs level impedes excitatory and inhibitory neurotransmitters release which affects immune homeostasis and energy balance while increasing gut permeability (59, 60). Direct evidence of intestinal microbe-mediated eCB system manipulation comes from a recent study where *Candida albicans* manifestation altered the levels of lipid and eCBs in the brain and gastrointestinal (GI) tract leading to increased anxiety-like behavior in mice (61). Considering the relevance of eCB system and gut microbiota in the manipulation of the immune system, it is inevitable to explore the possible relationships and mechanisms between both systems from the perspective of inflammatory diseases.

## Alteration of the Gut Microbiota by Cannabinoids

Various lines of ongoing research have connected the gut microbiota with metabolic and neurological disorders (52). Dietary interventions with specific fatty acids have been reported to increase the level of eCBs in human observational studies. These changes in eCBs have been attributed to variations in Peptostreptococcaceae, Veillonellaceae, and Akkermansiaceae (62). Cannabis consumption has been demonstrated to alter eCB tone and induce mucosal healing in ulcerative colitis (UC) patients in addition to improving quality of life (63). Modulation of eCB system using cannabinoids has been demonstrated to favor immune suppression *in vivo* (64). Present-day research targets to unravel the role of exogenous as well as endogenous cannabinoids in gut microbiota modulations and their impact on neurological and inflammatory conditions. Our lab has published multiple research articles on the alterations of microbiome and inflammation employing endogenous and exogenous cannabinoids (64, 65). A recent study demonstrated that the eCB, AEA, reversed the adverse microbiota perturbations instigated by SEB-mediated ARDS in mice. AEA treatment increased the abundance of beneficial bacteria producing SCFAs such as butyrate. In addition, AEA treatment curbed inflammation in the lungs and in the gut-associated mesenteric lymph node (MLN). Production of antimicrobial peptides (AMPs) and tight junction proteins (TJPs) which are key molecules sustaining epithelial barrier integrity in lung epithelial cells, as validated by single-cell RNA (Sc-RNA) sequencing were reported to attenuate the inflammation. Also, in this study, pathogenic Enterobacteriaceae and *Pseudomonas* were seen in the lungs of mice with ARDS while treatment with AEA led to their disappearance. Furthermore, the relative abundance of butyrate producing Lachnospiraceae and Clostridia were enhanced with AEA treatment (64). Emphasizing this observation, the abundance of butyrate-producing Firmicutes compared to *Bacteroides* was discovered following THC treatment of mice with diet-induced obesity (DIO) (66). In a similar line of study, the efficacy of THC to ease SEB-induced ARDS was examined. THC treatment improved the abundance of beneficial bacteria, *Ruminococcus gnavus*, while reducing pathogenic *Akkermansia muciniphila* in the lungs and gut. THC administration enriched SCFAs, specifically propionic acid, which attenuated the inflammatory response and protected mice from fatality. This study concluded that THC-induced reversal of microbial dysbiosis played a central role in the diminution of SEB-induced ARDS (65).

Colitis is another noteworthy disease model where the influence of cannabinoids on microbiota has been effectively demonstrated (67, 68). One study from our lab explored the effects of CBR activation following administration of THC and CBD either alone or in combination, in a chemically-induced murine colitis model. THC improved colonic barrier integrity as a result of higher mucus, AMPs, and TJPs production. Albeit alteration of the gut microbiota towards gram-negative bacteria was observed, the authors noted that the favorable effects of THC were not associated with microbiome modulation (67). A recent study presented the synergistic effect of fish oil and CBD treatment in the murine model of colitis. Co-administration of fish oil and CBD reduced inflammatory markers and ameliorated intestinal permeability in dextran sulfate sodium (DSS) model of mouse colitis. However, independent treatment with either of these failed to generate a favorable effect. The colonic inflammation was alleviated independent of the increased abundance of *A. muciniphila*. Of note is that the combination therapy reduced the abundance of Marinifilaceae, Desulfovibrionaceae, and Ruminococcaceae. Interestingly, Desulfovibrionaceae abundance has been reported in IBD and UC patients suggesting the functional role these microbial families play in GI diseases (68, 69). Another study investigated the role of gut microbiota in tempering clinical symptoms of paralysis and inflammation following cannabinoids treatment in an experimental autoimmune encephalomyelitis (EAE), a mouse model of MS. A combination of THC and CBD alleviated the symptoms of EAE and decreased pro-inflammatory cytokines while enhancing anti-inflammatory cytokine production. The EAE disease model showed abundant mucin degrading *A. muciniphila* which was considerably decreased following treatment with THC and CBD. A higher level of LPS was found in the brains of EAE mice while this scenario was reversed with cannabinoids treatment (70). The potential of cannabis extract to improve gut barrier function was investigated in the poultry industry where necrotic enteritis caused by *Clostridium perfringens* caused mortality in birds leading to economic loss along with the potential hazard of pathogen transmission to the consumer via the food chain. A combination of cannabis extract and selenium nanoparticles altered the response of chickens towards *C. perfringens*. This treatment upregulated the expression of genes involved in gut barrier function and improved collagenase activity. However, the extract alone could not generate significant beneficial effects (71).

Synthetic cannabinoids have also been extensively studied for their anti-inflammatory properties and their ability to alter the gut microbiota. Treatment with the CB2R agonist, JWH133 alleviated overgrowth of bacteria, bacterial translocation, and bacterial peritonitis, up-regulated intestinal TJPs, and reduced intestinal oxidative stress in cirrhotic rats. Furthermore, the treatment considerably diminished the levels of TNFα and inflammatory facilitators, intestinal mucosal impairment, and infection (72). Blockade of CB1R using the antagonist, Rimonabant reduced DIO and inflammatory cytokines. Trafficking of M1 macrophages and decreased intestinal permeability were also observed with CB1R blocking. Further metagenomics analysis demonstrated an elevated relative abundance of *A. muciniphila* and reduced abundance of Lanchnospiraceae and Erysipelotrichaceae in the gut (73). Nabilone, a CB1R agonist, was found useful in the treatment of post-traumatic stress disorder (PTSD), nausea, and vomiting associated with chemotherapy and pain management (74, 75). Administration of nabilone for 3 months improved health and alleviated diarrheal symptoms in patients. Although microbial dysbiosis was not investigated in this study, it encourages further clinically-oriented investigations on the effect of cannabinoids on such disease models as related to microbial dysbiosis (76).

## Immunomodulatory Mechanisms of Gut Microbiota

Endogenous, as well as exogenous cannabinoids, have been widely recognized to regulate inflammation and mucosal permeability of the GI tract where they possibly interact with the gut microbiome. In this section, we have tried to pull together the known mechanisms through which cannabinoids control microbial dysbiosis and accompanying inflammation. The indispensable role of gut microbiota on immune regulation has been excellently validated by multiple investigations. For example, one such study demonstrated the ability of commensal segmented filamentous bacterium (SFB) to induce CD4^+^ T cells to produce IL-17 and IL-22 in the lamina propria of mice. SFB adhered to the Th17 cells and induced inflammation and production of antimicrobial defensins (77). In the gut, under normal circumstances, eCB system is regulated by CB1R. However, both CB1R and CB2R get activated during inflammation leading to anti-inflammatory cytokine production that suppresses inflammation and intestinal damage (78).

The crosstalk between eCB system and gut microbiota has been established with murine models of obesity where low-grade inflammation and increased eCB system tone are reported. Obese mice exhibited higher colonic CB1R colonic mRNA and the modulation of gut microbiota with the use of prebiotics reduced this scenario. Moreover, prebiotic treatment alleviated CB1R mRNA and concentration of AEA in genetically obese mice explaining the involvement of the gut microbial community on CB1R and eCB expression. The same study disclosed the maintenance of intestinal barrier integrity by eCB system (79). Reduction in the number of TJPs increases the space between epithelial cells promoting paracellular translocation of microbial metabolites from the intestinal lumen to circulation and other organs, and elevated LPS levels impair adipogenesis and promote inflammation (79). THC administration has been reported to reduce LPS levels in mice while increasing TJPs. THC mediated CBR modulation reduced plasma LPS levels by altering the distribution and localization of TJPs which led to improved gut barrier function (65, 70).

Microbial-derived SCFAs, neurotransmitters, and amino acids take part in the immune, endocrinal, and neuronal signaling pathways via binding to host receptors (80, 81). Multiple investigations have validated the potential of AEA and THC to enhance the levels of SCFAs and AMPs in murine models of inflammation (64–67). SCFAs are produced in the colon by fermentation and subsequent degradation of undigested dietary fibers by gut harboring bacteria and they contribute to the regulation of both innate and adaptive immunity of the host. Acetate and propionate, produced by Bacteroidetes, and butyrate, produced by Firmicutes are the major SCFAs involved in host-bacterial communications (82). Blocking of histone deacetylases (HDAC) and activation of GPCRs are two main signaling pathways modulated by microbial SCFAs (80–82). Interestingly, GPCRs such as GPR43 and GPR109A are expressed by adipose tissue macrophages and dendritic cells (DCs). The binding of SCFAs to these receptors induces K^+^ efflux and membrane hyperpolarization which in turn stimulates NLRP3 inflammasome in primed macrophages to produce IL-18 (83, 84). Butyrate-dependent activation of GPR109A induces apoptosis of colon cancer cells. Furthermore, these receptor/ligand complexes inhibit nuclear factor-kappaB (NF-κB) activation in the colon of mice (85). Butyrate enhanced the function of human TGFβ1 in the intestinal epithelial cells (IECs) which in turn directed the accumulation of Treg cells in the lumen, and the study suggested inhibition of HDAC as the major mechanism behind this activity. Butyrate-induced HDAC inhibition down-regulated the generation of LPS-triggered pro-inflammatory cytokines such as IL-6 and IL-12 (86, 87). Another study demonstrated that butyrate enhanced the expression of AMPs, LL-37, and CAP-18 by IECs in rabbits (88). In a similar manner, activation of genes encoding host defense peptides in HD11 macrophages and monocytes has been observed in chickens following butyrate consumption (89). Succinate, another SCFA produced by the gut bacteria, *Prevotella copri* was shown to be involved in gut gluconeogenesis and improved glucose homeostasis (90). Once transported into circulation, SCFAs exert their effect on distant organs as well. For example, circulating propionate modified bone marrow hematopoiesis by increasing levels of macrophages and DCs precursors. The phagocytic DCs invaded the lung but lacked Th2 effector cell differentiation ability and controlled inflammation (91). SCFAs, as evident from numerous studies, represent the most important connecting link between gut microbiome and host immune homeostasis.

**FIGURE 1:**
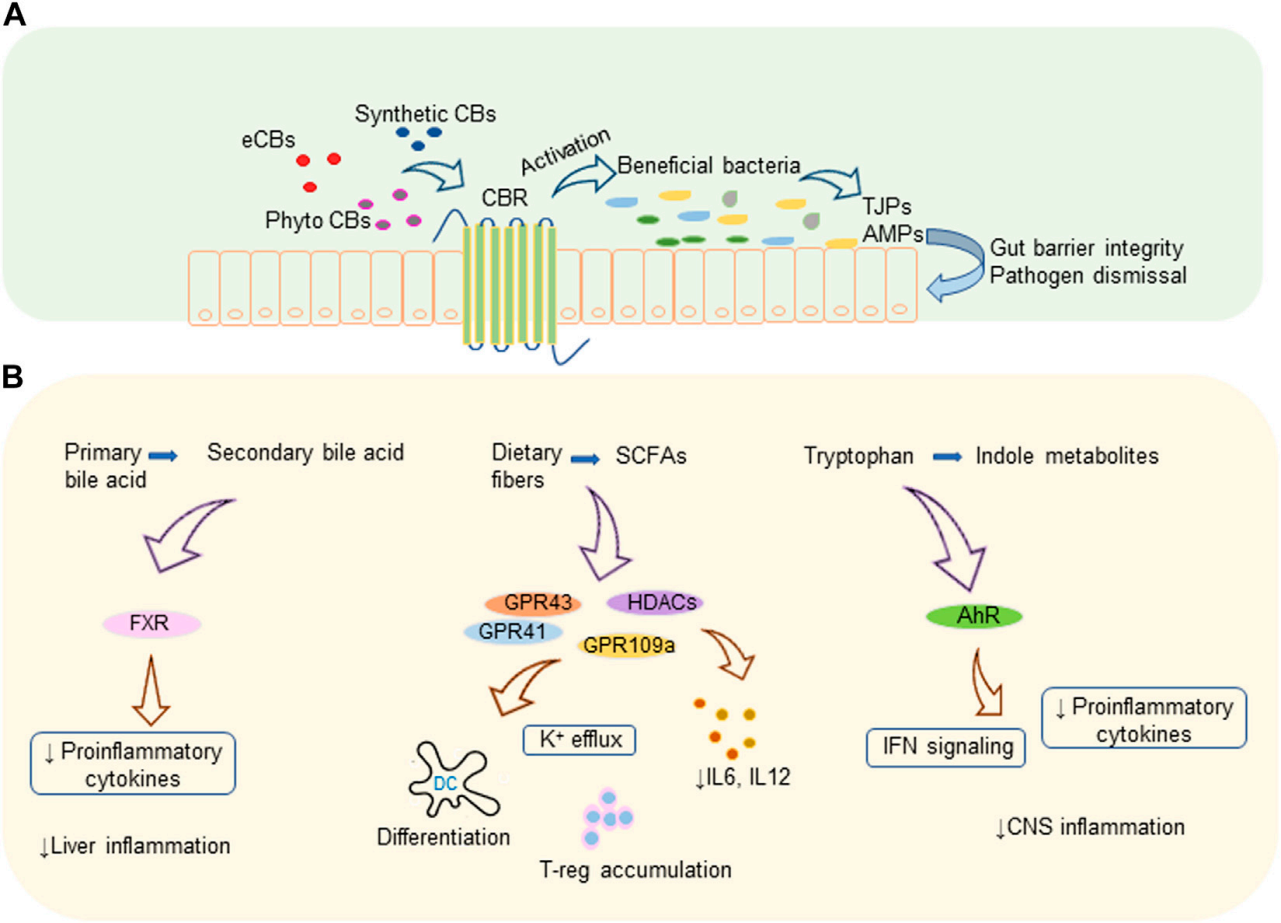
Cannabinoids and gut microbiota. **(A)** Cannabinoid mediated microbiome modulation: endogenous or exogenous cannabinoids increase the beneficial bacteria which produce TJPs that improve gut barrier integrity and AMPs that eliminate pathogens. **(B)** Immunomodulatory mechanisms of microbial metabolites: microbiota generated secondary bile acids, SCFAs, and indole metabolites modulate various receptors leading to decreased pro-inflammatory cytokines and immune suppression. AhR, aryl hydrocarbon receptor; AMP, antimicrobial protein; CBR, cannabinoid receptor; CBs, cannabinoids; CNS, central nervous system; eCBs, endocannabinoids; FXR, farnesoid X receptor; GPR, G-protein-coupled receptors; HDACs, histone deacetylases; IFN, interferon; IL, interleukin; K, potassium; TJP, tight junction proteins; T-reg, regulatory T cell.

Bile acid metabolism is another activity implemented by a variety of gut microbes harboring the gut. Microbes convert primary bile acid to secondary and tertiary bile acids *via* various mechanisms that include deconjugation of glycine and taurine by bile salt hydrolase, de-hydroxylation as well as dehydrogenation and epimerization of cholesterol core (92). Members of the genera *Bifidobacterium*, *Clostridium*, and *Lactobacillus* are reported to efficiently metabolize primary bile acids (92, 93). Secondary bile acid metabolism and prevention of bile acid production in the liver by activating nuclear receptor farnesoid X receptor (FXR) in the ileum by gut microbiota controls liver inflammation. Also, intestinal microbiota decreases the levels of pro-inflammatory cytokines which are involved in reducing the transcription of FXR target genes (94). Proteins and peptides in the diets are digested to free amino acids as a result of microbial fermentation and major amino acid of such kind is tryptophan. Tryptophan metabolites are another set of biologically active metabolites generated by intestinal bacteria that affect intestinal epithelial barrier integrity as well as the organogenesis of intestinal lymphoid follicles. Members of the phylum Firmicutes convert tryptophan to tryptamine and other indole derivatives (95, 96). A recent study showed that tryptamine can attenuate neuroinflammation in the murine model of MS (97). *Lactobacillus* strains were found to efficiently metabolize Tryptophan to its derivatives which act as aryl hydrocarbon receptor (AhR) ligands in the colitis mouse model (98). These metabolites, mostly indoles, act as AhR agonists and regulate type-1 IFN signaling in astrocytes leading to suppression of central nervous system (CNS) inflammation (99). AhR signaling mediates IL-22 production in the gut by activating innate lymphoid cell 3 (ILC3) (100). In addition, AhR has been shown to play a vital role in the development of ILC and intraepithelial lymphocytes (100, 101). How AhR activation leads to suppression of inflammation has been the topic of recent reviews (102, 103). Figure 1 illustrates a summary of cannabinoid mediated microbiome modulation and the immunomodulatory mechanism of microbial metabolites.

While most of the studies that we have reviewed above have shown an association between the administration of cannabinoids and suppression of inflammation to changes in the microbiota, one can question whether these studies merely indicate a relationship between these events or whether the cannabinoid-mediated alterations in the microbiota are actually responsible for inducing attenuation of inflammation. The association between microbial changes seen following exposure to cannabinoids and the consequent impact of such changes in immunomodulation can only be proven through fecal microbiota transplants (FMT).

There is evidence to suggest the role of microbiota on eCB signaling through use of FMT. Multiple studies reported that FMT-mediated microbial dysbiosis can modify eCB signaling (104, 105). One study clearly investigated the impact of FMT from conventionally raised mice to germ-free mice. Endocannabinoidome gene expression and lipidomics were analyzed by transcriptomics and LC-MS/MS before and after FMT. Age-dependent endocannabinoidome gene expression and lipid variations in the germ-free mice were reversed following FMT from age-matched conventionally raised donor mice (106). In another study, FMT from murine models of EAE disease treated with THC and CBD, into antibiotics treated, microbe depleted mice demonstrated that the recipient mice showed decreased EAE disease severity (70). A similar kind of study was conducted in a murine model of SEB-mediated ARDS. The microbiota transplanted from THC-treated ARDS mice into antibiotic-treated, microbiome-depleted recipient mice showed better survival from ARDS than those that received FMT from the control group. FMT from THC-treated groups caused a decrease in inflammatory CD4^+^ and CD8^+^ T cells and an increase in immune suppressive MDSCs and Tregs in the lungs (65). Such studies clearly demonstrate that endogenous and exogenous cannabinoids can promote beneficial microbiota in the gut that can attenuate inflammatory diseases even in distal organs.

## Conclusion

The communications among eCB system, immune regulation, and gut microbiota are intricately interconnected. CBRs agonists/antagonists have been pre-clinically validated to be useful in the treatment of metabolic conditions, such as obesity and diabetes as well as in disease models of colitis and cardiometabolic malfunctions. Also, well-established is the role of intestinal microbial community in the onset or progression of these disorders. The numerous groups of microbial clusters and the myriad of biologically active metabolites produced by them along with their receptors trigger extensive signaling pathways that affect the energy balance and immune homeostasis of the host. The microbiome-eCB signaling modulation exploiting exo- or endogenous cannabinoids opens a new avenue of cannabinoid-gut microbiota-based therapeutics to curb metabolic and immune-oriented conditions. However, more clinical investigations are essential to validate this concept.

## Glossary

2-AG: 2-arachydonoylglycerol
AEA: anandamide
AhR: aryl hydrocarbon receptor
AMPs: antimicrobial peptides
ARDS: acute respiratory distress syndrome
cAMP: cyclic adenosine monophosphate
CBD: cannabidiol
CNS: central nervous system
CBR: cannabinoid receptors
DCs: dendritic cells
DIO: diet-induced obesity
DNMT: DNA methyl transferases
DSS: dextran sulfate sodium
EAE: experimental autoimmune encephalomyelitis
ERK: extracellular signal-regulated kinase
FAAH: fatty acid amide hydrolase
FAK: focal adhesion kinase
FMT: fecal microbiota transplants
FXR: farnesoid X receptor
GPCRs: G-protein-coupled receptors
GI: gastrointestinal
HDAC: histone deacetylase
IBD: inflammatory bowel disease
IECs: intestinal epithelial cells
IFN-γ: interferon-gamma
IL: interleukin
ILC3: innate lymphoid cell 3
LPS: lipopolysaccharides
MDSCs: myeloid-derived suppressor cells
miRNAs: microRNAs
MLN: mesenteric lymph node
MS: multiple sclerosis
PI3K-PKB/AKT: phosphoinositide-3-kinase–protein kinase B/Akt
p38 MAP: p38 mitogen-activated protein
PTSD: post-traumatic stress disorder
SCFAs: short-chain fatty acids
Sc-RNA: single-cell RNA
SEB: staphylococcal enterotoxin B
SFB: segmented filamentous bacterium
TGF-β: transforming growth factor β
Th1: T helper 1
Th2: T helper 2
THC: δ9-tertrahydrocannabinol
TJPs: tight junction proteins
Tregs: regulatory T cells
TRPV1: transient receptor potential vanilloid 1
UC: ulcerative colitis
